# Phospholipase A2 receptor-negative membranous nephropathy presenting as a rare renal manifestation of IgG4-related disease

**DOI:** 10.1177/2050313X241279696

**Published:** 2024-08-31

**Authors:** Srikar Sama, Alexander Weickhardt, Preethi Subramanian, Pooja Reddy

**Affiliations:** 1 Department of Internal Medicine, University of California San Francisco Fresno, Fresno, CA, USA; 2 Department of Anatomic and Clinical Pathology, University of California San Francisco, San Francisco, CA, USA; 3 Department of Nephrology, Veteran Affairs Central California Health System, Fresno, CA, USA; 4 Department of Rheumatology, Veteran Affairs Central California Health System, Fresno, CA, USA

**Keywords:** IgG4-related disease, secondary membranous nephropathy, PLA2R-negative, rituximab, tubulointerstitial nephritis

## Abstract

IgG4-related disease is a fibroinflammatory condition characterized by dense lymphoplasmacytic infiltrates rich in IgG4-positive plasma cells affecting multiple organs. Though the most common renal manifestation of IgG4-related disease is tubulointerstitial nephritis, it can rarely present as secondary membranous nephropathy. We present a case of a 75-year-old male with phospholipase A2 receptor-negative membranous nephropathy as an atypical manifestation of IgG4-related disease. The patient presented with nephrotic syndrome and was found to have elevated serum IgG4 levels and IgG4-positive plasma cells in the kidney biopsy. He was successfully treated with corticosteroids and rituximab, resulting in significant improvement in proteinuria and normalization of IgG4 levels. This case highlights the importance of considering IgG4-related disease in patients with phospholipase A2 receptor-negative membranous nephropathy, especially in those with a history of other organ involvement. Early recognition and treatment of IgG4-related disease are crucial to prevent progressive kidney damage and improve patient outcomes.

## Introduction

IgG4-related disease (IgG4-RD) is a systemic fibroinflammatory condition characterized by mass-forming lesions with lymphoplasmacytic infiltrates that are rich in IgG4-positive plasma cells and elevated IgG4 levels.^1^ While it can affect various organs, renal involvement is not uncommon. The most common renal manifestation of IgG4-RD is tubulointerstitial nephritis (TIN).^2^ However, glomerular disease is also a possible occurrence, although it is much less common than TIN. Membranous glomerulonephritis (MGN) or membranous nephropathy (MN) is the most frequently reported glomerular disease in IgG4-RD. MN can occur alone or simultaneously with IgG4-related TIN, as described in a two-biopsy series of IgG4-related TIN where ~7% (4/58) of patients had MN.^3^ IgG4-related MN affects men more than women in contrast to many other autoimmune diseases, and the mean age of patients is 58–65 years.^3,4^ In this case, we describe a 75-year-old male who presented with PLA2R-negative MN, which is a less-recognized renal manifestation of IgG4-RD.

## Case

A 75-year-old male with a past medical history of acoustic neuroma, hypothyroidism, benign pancreatic mass status-post surgery with partial pancreatectomy and splenectomy (Pathology unavailable) presented to the nephrology clinic for nephrotic range proteinuria, bilateral lower extremity swelling, and weight gain over the past 3 months which was refractory to loop diuretics. His physical exam was remarkable for 3+ pitting edema in all dependent areas of the body.

Laboratory tests revealed nephrotic range proteinuria (protein excretion 12 g/24 h; reference range <150 mg/24 h), urine albumin/creatinine ratio: >300 µg/mg (<30 µg/mg), decreased estimated glomerular filtration rate: 48 ml/min (>60 ml/min), and eosinophilia (absolute eosinophil count 800 cells/µL (Patient’s baseline: 500 cells/µL, reference <330 cells/µL)). Tests for autoimmune conditions (ANA, dsDNA, cryoglobulins) and infectious etiologies (hepatitis, HIV, RPR, TB, and coccidioidomycosis) were negative. C-reactive protein was normal (<0.5 mg/dL; 1.5–3 mg/dL). Serum protein electrophoresis complement studies revealed low C4: 8 mg/dL (15–53 mg/dL) and borderline low C3: 79 mg/dL (82–185 mg/dL). Serum protein electrophoresis revealed a relative increase of beta-2 globulins: 1.1 g/dl (0.2–0.5 g/dl) to beta-1 globulins: 0.3 g/dl (0.4–0.6 g/dl), and the follow-up immunofixation was negative for monoclonal proteins. Imaging studies, including ultrasound and computed tomography scans of the kidneys, were unremarkable, showing no abnormalities.

**Figure 1.**
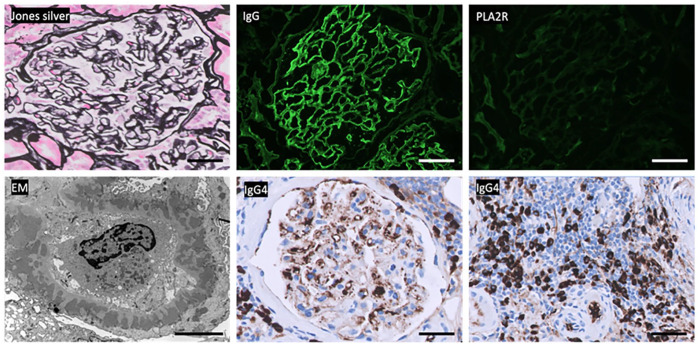
Diagnostic features of phospholipase A2 receptor (PLA2R)-negative membranous glomerulonephritis: glomerular basement membrane thickening (Jones silver stain), IgG+ granular peripheral capillary loop immune deposits (IgG immunofluorescence), negative PLA2R (PLA2R immunofluorescence), and subepithelial electron-dense deposits with capillary wall remodeling (electron microscopy (EM)). Additional features of IgG4-related renal disease: glomerular IgG+ deposits and tubulointerstitial IgG+ plasma cells (IgG4 immunoperoxidase stain). EM: 4800× magnification, 5 µm scale bar. Other images: 400× magnification, 50 µm scale bars.

A kidney biopsy (27 glomeruli, 9 of which were sclerotic) showed thickening of glomerular capillary walls and moderate patchy interstitial fibrosis and tubular atrophy (30%). No mesangial hypercellularity or mesangial matrix accumulation was seen. Immunofluorescence and electron microscopy were diagnostic of phospholipase A2 receptor (PLA2R) negative membranous glomerulonephritis (MN) with moderate chronicity (Figure 1). PLA2R negativity is not in favor of “primary” (autoimmune) etiology; however, other primary etiologies mediated by autoantibodies against antigens including thrombospondin type-1 domain-containing 7A, neural epidermal growth factor-like 1, semaphorin-3 b, neural cell adhesion molecule 1, high-temperature recombinant protein Al, and protocadherin 7 were negative. Exostatin1/exostatin2 (EXT1/EXT2) showed weak staining, which was reported as equivocal. Given the history of the pancreatic mass and MN, IgG levels were tested via western blot revealing elevated IgG: 2517 mg/dL (600–1540 mg/dL) with a significant increase in the IgG4 subclass: 1139 mg/dL (4–86 mg/dL). Immunoperoxidase evaluation of the biopsy also returned a few days later, showing IgG4-positive tubulointerstitial plasma cells in fibrotic areas. IgG4-RD typically manifests as TIN, but no features of active TIN were seen in this biopsy 2.

The patient was started on prednisone 40 mg with an excellent response but soon had a relapse with increased proteinuria while tapering steroid doses. He was subsequently initiated on rituximab infusions 1 g for two doses spaced 2 weeks apart, alongside a prednisone taper and showed excellent response by improvement in proteinuria and urine protein/creatinine ratio. There was a significant reduction in IgG4 levels (1139 mg/dL to 114 mg/dL), eosinophilia resolved, and complement levels normalized (Table 1). The patient is currently being followed closely by rheumatology and nephrology. He is receiving rituximab maintenance infusions of 1 g every 6 months, and the disease has been well controlled with this treatment.

**Table 1.** Laboratory Values at Different Stages of Treatment.

Test	Reference range	At the time of diagnosis	After steroids	Relapse	After rituximab
Urine albumin/creatinine (µg/mg)	<30 µg/mg	Too high to measure	2261 µg/mg	4270 µg/mg	985 µg/mg
Urine Protein/Creatinine (g/g)	<0.2 g/g	6.5 g/g	2.9 g/g	8.12 g/g	1.51 g/g
Estimated Glomerular Filtration Rate (eGFR, ml/min/1.73 m²)	>60 ml/min/1.73 m²	48 ml/min/1.73 m²	63 ml/min/1.73 m²	39 ml/min/1.73 m²	63 ml/min/1.73 m²
Creatinine (mg/dL)	0.6–1.2 mg/dL	1.5 mg/dL	1.2 mg/dL	1.4 mg/dL	1.2 mg/dL
Serum IgG4 (mg/dL)	4–86 mg/dL	1139 mg/dL	391 mg/dL	378 mg/dL	114 mg/dL
Complement C3 (mg/dL)	82–185 mg/dL	79 mg/dL	133 mg/dL	97 mg/dL	138 mg/dL
Complement C4 (mg/dL)	17–53 mg/dL	9 mg/dL	30 mg/dL	22 mg/dL	27 mg/dL
Eosinophils (cell/µL)	<500cell/µL	800 cell/µL	60cell/µL	120 cell/µL	70 cell/µL

This table summarizes the laboratory values at four different stages, from left to right: at the time of diagnosis, after steroid treatment, at relapse, and after rituximab treatment.

## Discussion

MN is a type of glomerular injury that may be primary or secondary. Almost 75% of MN are primary MN, and most of them involve antibodies against a podocyte antigen, PLA2R.^3^ Secondary MN can be attributed to various etiologic factors, including autoimmune diseases, infections, malignancies, and medications.^5^ IgG4-RD is a well-recognized fibroinflammatory condition affecting multiple organs, including the kidneys. TIN is the most common renal manifestation of IgG4-RD.^1–5^ However, IgG4-RD can also present as secondary MN as described in a nine case series study conducted by Alexander et al.,^5^ but the incidence is less and is only seen in approximately 10%–16% of IgG4-related kidney disease.^6,7^ A recent cohort study conducted by the Mayo Clinic has identified that a subset (7%) of IgG4-related tubulointerstitial nephritis (IgG4-TIN) cases present with an acute interstitial nephritis pattern.^8^ This finding highlights a previously unrecognized manifestation of IgG4-TIN, broadening the understanding of its renal involvement.

A retrospective cohort study by Qi et al.^9^ demonstrates that serum IgG4 test sensitivity is significantly higher in Asian patients (96%) compared to non-Asians (67%). Additionally, median serum IgG4 levels at diagnosis are elevated in Asians (11.2 g/L) versus non-Asians (2.9 g/L). The patient in our case report is White, with an IgG4 level of 1.1 g at diagnosis, aligning with this study and underscoring the importance of considering racial differences in the diagnostic evaluation of IgG4-RD.

IgG4-RD is a recognized multiorgan disease that is associated with secondary MN.^5^ While TIN is the most common kidney manifestation of IgG4-RD,^1–5^ it can also be related to secondary MN, as described in a nine case series study by Alexander et al.^5^ Anti-PLA2R antibodies help differentiate primary from secondary MN with a sensitivity of 74%.^10,11^ Our patient stained negative for PLA2R and fit the comprehensive diagnostic criteria for IgG4-RD proposed by a Japanese research team.^12^ This includes nephrotic range proteinuria with hypocomplementemia, elevated serum IgG4 level, and pathology showing dense lymphoplasmacytic infiltration by IgG4-positive plasma cells with fibrosis surrounding plasma cells. Complement levels may or may not be low, as seen in the case of series by Alexander et al.;^5^ only one out of six patients had low complements.

IgG4-related MN can mimic lupus nephritis with positive ANA, dsDNA, and full house staining on kidney biopsy.^13^ Differentiation is challenging but crucial because of the difference in treatment and prognosis. Interestingly, two case reports show an association between MN and benign nerve sheath tumors. In both studies, MN was remitted completely after the excision of the tumor.^14,15^ The patient being discussed has an acoustic neuroma, but it was not resected. Additionally, many cases of MN have also been associated with COVID-19 vaccination, as reported by Ma et al.,^16^ which our patient received in 2021. However, further research is needed to establish causality in both of these associations.

Ig4-RD usually responds well to glucocorticoids, making it a popular first-line treatment option. However, while many patients initially respond positively to glucocorticoid treatment, the disease often relapses during tapering, requiring repeated courses of treatment to maintain remission.^17^ Patients who fail glucocorticoid monotherapy or patients with extremely high serum IgG4 usually require another agent like rituximab alongside glucocorticoids. Rituximab is shown to be effective in both induction therapy and the treatment of relapses in IgG4-RD and is associated with longer relapse-free survival.^18–20^

## Conclusion

PLA2R-negative MN can be a diagnostic challenge, and IgG4-RD should be considered in the differential diagnosis. In patients with MN who stain negative for PLA2R and have suggestive features, a high index of suspicion should be maintained, and the possibility of IgG4-related kidney disease should be carefully assessed. Prompt recognition and treatment of IgG4-RD are essential to prevent progressive kidney damage and improve patient outcomes. Further research is needed to elucidate the mechanisms underlying IgG4-RD-related MN and to optimize treatment strategies for this condition.
